# Natural killer T cells in allergic asthma: implications for the development of novel immunotherapeutical strategies

**DOI:** 10.3389/fimmu.2024.1364774

**Published:** 2024-04-02

**Authors:** Cristián Gutiérrez-Vera, Richard García-Betancourt, Pablo A. Palacios, Marioly Müller, David A. Montero, Carlos Verdugo, Francisca Ortiz, Felipe Simon, Alexis M. Kalergis, Pablo A. González, Noemi A. Saavedra-Avila, Steven A. Porcelli, Leandro J. Carreño

**Affiliations:** ^1^ Millennium Institute on Immunology and Immunotherapy, Santiago, Chile; ^2^ Programa de Inmunología, Instituto de Ciencias Biomédicas, Facultad de Medicina, Universidad de Chile, Santiago, Chile; ^3^ Departamento de Tecnología Médica, Facultad de Medicina, Universidad de Chile, Santiago, Chile; ^4^ Facultad de Ciencias de la Vida, Universidad Andrés Bello, Santiago, Chile; ^5^ Facultad de Ciencias Biológicas, Pontificia Universidad Católica de Chile, Santiago, Chile; ^6^ Department of Microbiology and Immunology, Albert Einstein College of Medicine, Bronx, NY, United States

**Keywords:** natural killer T cells, allergic diseases, asthma, immunomodulation, immunotherapy

## Abstract

Allergic asthma has emerged as a prevalent allergic disease worldwide, affecting most prominently both young individuals and lower-income populations in developing and developed countries. To devise effective and curative immunotherapy, it is crucial to comprehend the intricate nature of this condition, characterized by an immune response imbalance that favors a proinflammatory profile orchestrated by diverse subsets of immune cells. Although the involvement of Natural Killer T (NKT) cells in asthma pathology is frequently implied, their specific contributions to disease onset and progression remain incompletely understood. Given their remarkable ability to modulate the immune response through the rapid secretion of various cytokines, NKT cells represent a promising target for the development of effective immunotherapy against allergic asthma. This review provides a comprehensive summary of the current understanding of NKT cells in the context of allergic asthma, along with novel therapeutic approaches that leverage the functional response of these cells.

## 1 Introduction

Asthma is one of the most common respiratory disorders, affecting more than 300 million children and adults and causing approximately 250,000 deaths each year (1, 2). This inflammatory disease is often a life-long chronic pulmonary disorder characterized by airway hyperresponsiveness and airflow obstruction, severely impacting patients’ quality of life. The prevalence of allergic diseases has increased worldwide over the last several decades. Currently, the annual economic burden of asthma in the United States is approximately 56 billion USD (3), while the estimated economic costs per patient range from 1,900 to 3,200 USD per year (4). Notwithstanding the aforementioned, different phenotypes of asthma have been defined, such as occupational, cigarette smoke-induced, air pollution-induced, and exercise-induced asthma, which lack the allergic response, mainly given by the functional response associated with immunoglobulin E (IgE) (5, 6).

Currently, the most common treatment for asthmatic disease is inhaled corticosteroids. However, this could eventually lead to steroid-refractory airway inflammation since airway remodeling effects due to asthma are not avoided (7). Additionally, uncontrolled comorbidities can increase the severity of asthma (8). Other pharmacological therapies that have emerged as promising curative treatments include systemic corticosteroids and novel immunotherapeutic-based strategies (9). Nonetheless, failure of patients to adhere to asthma treatments ranges from 30 to 70% (10). This adds to the fact that the palliative effect of some medications disappears when the drug is discontinued, and the airway remodeling changes are irreversible (11, 12). Thus, there is currently no cure for asthma, and its treatment focuses on improving its symptoms (8).

Within this context, understanding the cellular and molecular interactions that occur during the genesis and development of asthma, especially the pathological and protective roles played by different immune cells, is imperative to improve the effectiveness of the current immunotherapies. The first step is determining the presence, function, and interplay of the different immune cells involved in asthmatic disease.

Natural killer T (NKT) cells have been associated with a protective role in cancer and the development of autoimmune diseases (13). Importantly, different studies have shown contradictory results about the involvement of NKT cells in asthma (14, 15). In particular, it is still unclear whether these unconventional T cells have a pathological or protective role in the onset and development of asthma. Nevertheless, recent investigations in other pathologies, such as cancer, malaria, and HIV infection, have shown that stimulating NKT cells with different glycolipid antigens can modulate the immune response outcome to specific antigens with promising results (16). Hence, the modulation of the NKT cell functions to improve asthma-targeting immunotherapies could decrease airway obstruction by downregulating the inflammatory process and avoiding further damage to the pulmonary tissue, resulting in a novel therapeutic strategy.

In this review, we discuss the hallmarks of asthma, the current knowledge of NKT cell biology, and their role in allergic asthma. Additionally, we review recent findings in the function of NKT cells that might translate into their potential clinical applications sooner rather later.

## 2 Allergic asthma: general characteristics

### 2.1 Current definition of asthma and risk factors

**Figure 1:**
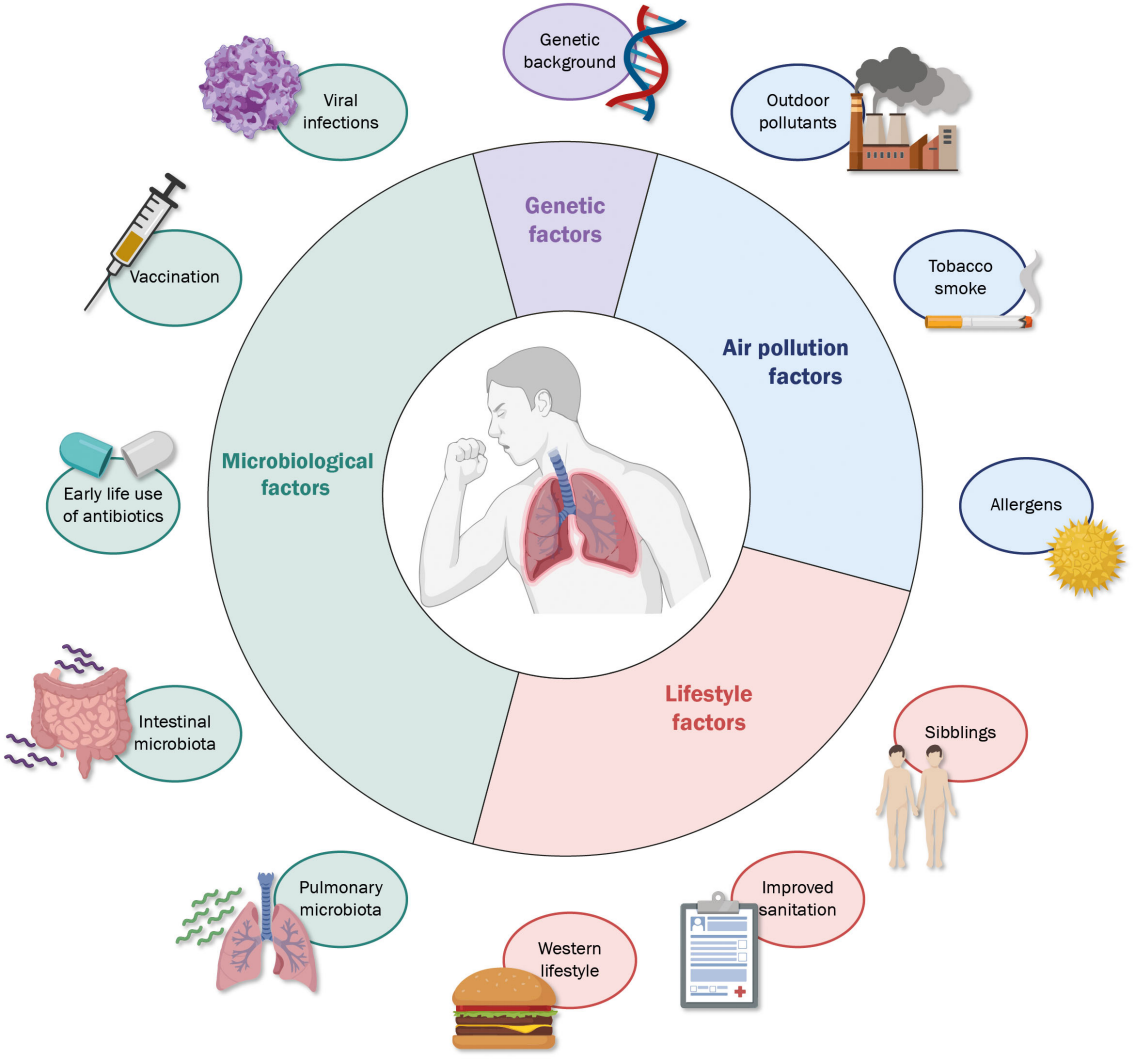
Different factors influence the development of allergic asthma. A wide range of characteristics, both related to the subject and surrounding environment, are determinants in the possible onset and degree of severity of allergic asthma, among which the most relevant include microbiological exposure (light green), genetic factors (light purple), exposure to environmental substances (light green) and factors to the environment and lifestyle (light red).

Asthma is a long-term respiratory disease characterized by chronic airway inflammation and symptoms such as wheezing, dyspnea, chest tightness, and cough; being considered a clinically heterogeneous disease with complex pathophysiology, and different factors may influence the development of asthma in susceptible individuals (**Figure 1**). Some asthma triggers include allergens, irritant substances, exercise, weather variation, and respiratory viruses (17). Airway hyperresponsiveness (AHR) is a crucial feature of this disease, and it is a consequence of a highly reactive response to innocuous foreign substances in asthmatic patients compared to healthy individuals (18). Additionally, airway inflammation leads to pulmonary dysfunction by releasing proinflammatory mediators that cause airway remodeling (19).

Asthma is clinically diagnosed by assessing different symptoms. These include recurrent wheezing, difficulty in breathing, chest tightness, occurring or worsening of the above symptoms at night, and occurrence of symptoms in the presence of exercise, viral infections, animal hair or fur, mold, and pollen, among other allergens (20). Other pathologies with similar symptoms, such as bronchiolitis, chronic obstructive pulmonary disease, cystic fibrosis, and chronic eosinophilic bronchitis, need to be excluded, and most symptoms should be reversed using a bronchodilator (21).

**Figure 2:**
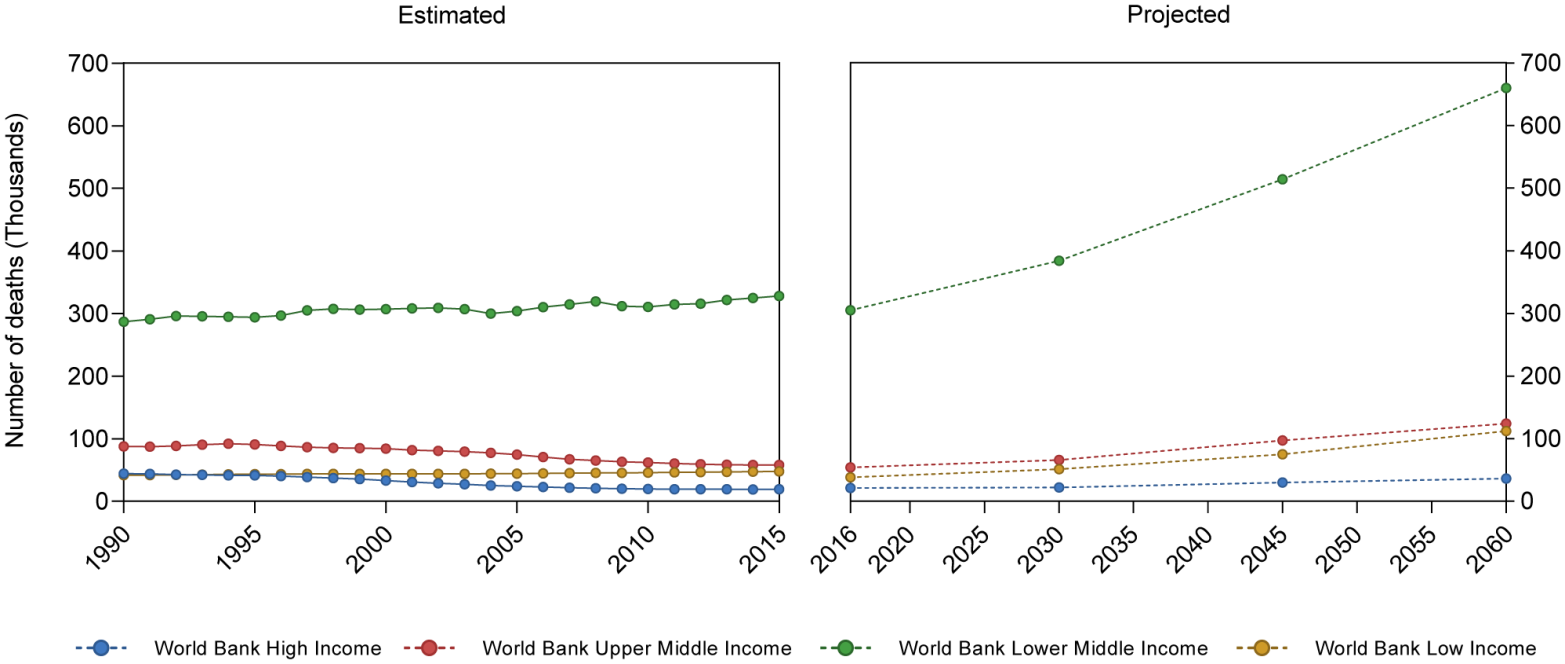
Asthma-related mean mortality reported and projected cases. Based on Institute for Health Metrics and Evaluation (IHME), GBD Results. (https://vizhub.healthdata.org/gbd-results/) and World Health Organization Global Health Estimates: Projection of deaths by cause, age and sex, (https://colinmathers.files.wordpress.com/2022/05/ghe_dthwbinc_proj_2016-2060.xlsx), categorizing countries according to World Bank income groups.

Asthmatic disease can develop at any age, although it is most common during childhood, and boys are more affected than girls, reversing in adulthood (22, 23). Geographical location also impacts asthma prevalence: countries such as Brazil, the Netherlands, United Kingdom, Sweden, and Australia, have the highest prevalence, ranging from 13% to 21.5% (24). On the other hand, on a global scale, low- and lower-middle-income countries present higher mortality rates in comparison to upper-middle and high-income countries (25) (**Figure 2**).

### 2.2 Origin, development and consequences of allergic asthma

**Figure 3:**
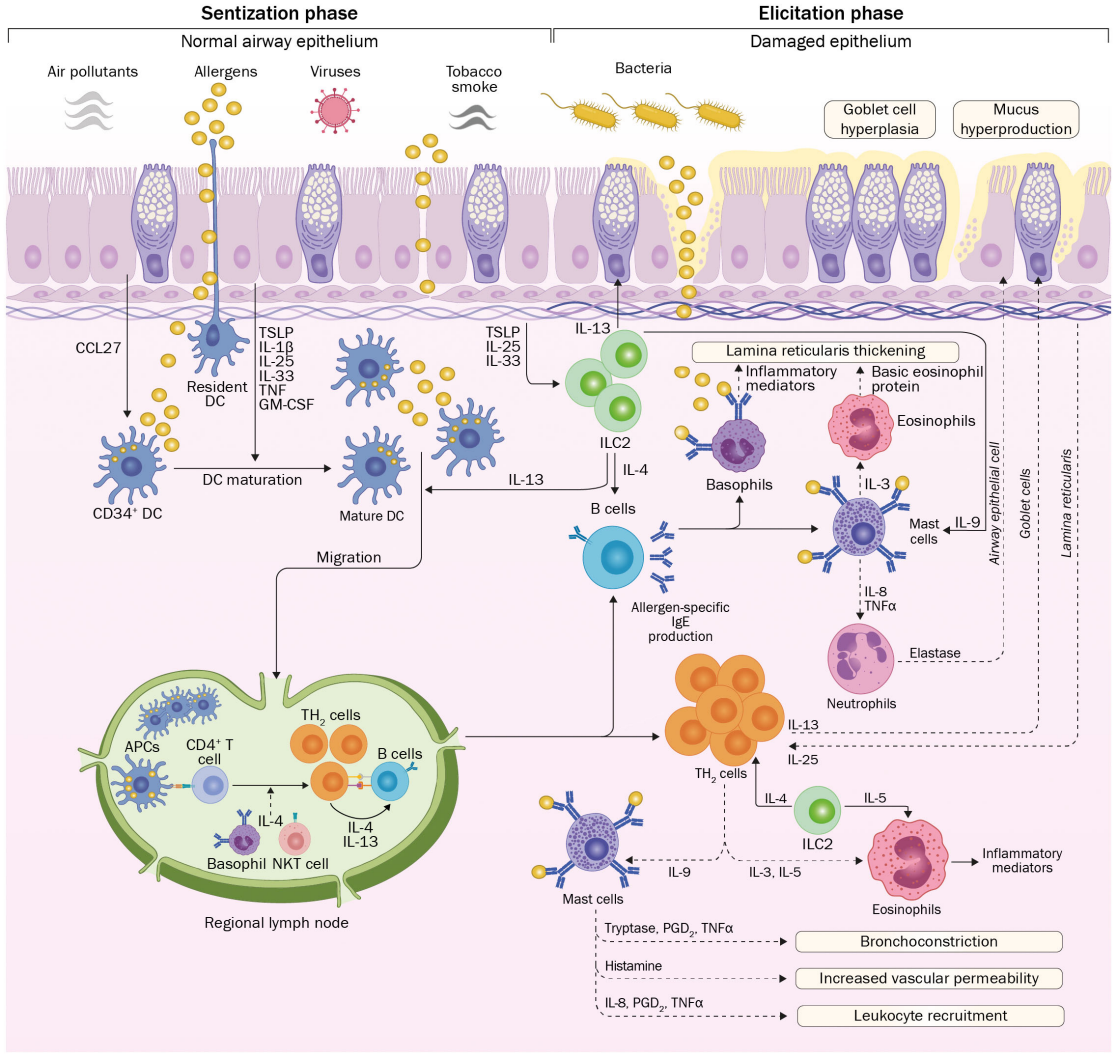
Onset and development of allergic asthma. Environmental allergens reach the airway epithelium, which could present different degrees of injuries due to exposure to irritating substances. Allergens are captured and processed by DCs with further migration to regional lymph nodes to present allergen-derived peptides to naïve T cells. Influenced by the cytokine milieu, differentiation to T_H_2 cells occurs. Further proliferation and return to the pulmonary epithelium, these cells produce a wide range of inflammatory cytokines, influencing the function of innate immune cells. In parallel, activation and isotype switching of B cells leads to the production of allergen-specific IgE. On posterior encounters of the epithelium with the allergen, innate immune responses are triggered by IgE-coated mast cells and basophils, leading to massive secretion of inflammatory cytokines that initiate the inflammatory process associated with allergic asthma. Repetitive exposure to asthma-inducing allergens will lead to tissue damage due to the continuous presence of inflammatory mediators, causing different modifications in the airway architecture. CCL27, C-C motif chemokine ligand 27; TSLP, Thymic stromal lymphopoietin; IL, interleukin; TNF, tumor necrosis factor; GM-CSF, Granulocyte-Macrophage Colony-Stimulating Factor; APC, antigen-presenting cell; Ig, immunoglobulin; PGD2, prostaglandin D2; T_H_2, T helper 2; ILC2: Innate lymphoid type 2 cell.

Allergic asthma onset requires exposure of the subject to allergens, which are defined as environmental substances [mites, molds, grass, trees, and weed pollens (**Table 1**)] that are innocuous for the majority of the population, but upon inhalation, ingestion or injection can lead to immediate IgE-mediated hypersensitivity in atopic subjects (41). After exposure, allergens trigger a T_H_2-cell response, characterized by interleukin (IL)-4 and IL-13 secretion, mediating B cell differentiation into immunoglobulin E (IgE)-producing cells (42). Later, in the elicitation phase, repeated exposure of the subject to the given allergen enhances allergen-specific IgE production and triggers the secretion of inflammatory cytokines by IgE-coated mast cells and basophils, which initiate airway remodeling (43, 44). These processes are depicted in **Figure 3** and discussed in detail below.

**Table 1:** Characterization of main allergens involved in allergic asthma.

Main source	Allergen	Biological function	Reference
Animals and arthropods			
House dust mite (*Dermatophagoides pteronyssinus*)	Der p 1	Cysteine and serine protease	(26)
	Der p 2	Lipid binding protein	(27)
	Der p 3	Trypsin-like serine protease	(28)
	Der p 5	Possible ligand-binding protein	(29)
Cat (*Felis domesticus*)	Fel d 1	Secretory globins	(30)
Dog (*Canis familiaris*)	Can f 1	Lipocalin	(31)
Mouse (*Mus musculus*)	Mus m 1	Lipocalin	(32)
Rat (*Rattus norvegicus*)	Rat n 1	Lipocalin	(33)
Cockroach (*Blattella germanica*)	Bla g 2	Inactive aspartic protease	(34)
Grasses			
Rye (*Lolium perenne*)	Lol p 1	Expansins	—
Timothy (*Phleum pratense*)	Phl p 5	Nucleases	(35)
Bermuda (*Cynodon dactylon*)	Cyn d 1	Expansins	—
Weeds			
Ragweed (*Artemisia artemisiifolia*)	Amb a 1	Pectate lyase	(36)
Trees			
Birch (*Betula verrucosa*)	Bet v 1	Pathogenesis-related protein	(37)
	Bet v 2	Profilin	(38)
Fungi			
*Aspergillus fumigatus*	Asp f 1	Cytotoxin	(39)
*Alternaria alternata*	Alt a 1	Possible role in plant pathogenesis	(40)

Initially, allergens reach the epithelium and penetrate different organs by disrupting their physical barrier (45) using protease activity (46) or by induction of immunological activity, mainly given by the secretion of thymic stromal lymphopoietin (TSLP) (47, 48). Allergens are captured and processed by dendritic cells (DCs) underneath the airway epithelium, which migrate to local lymph nodes and interact with naïve T cells (49). This event triggers naïve T cells proliferation and differentiation, which initiates sensitization and further allergen-specific responses (49). Since the subject is exposed to a complex mixture of molecules, including the given allergen, naïve CD4^+^ T cells differentiate into T-helper 2 (T_H_2)-lymphocytes (50). The respiratory epithelium also becomes actively involved in this process by secreting different cytokines, such as TSLP, TNF-α, IL-1β, IL-6, and IL-33, collectively referred to as alarmins (51). Particularly, TSLP, an IL-7-like cytokine, induces DCs maturation and skewing of the immune response toward a T_H_2 profile (52).

Upon activation, T_H_2 cells produce and secrete a wide range of cytokines that cause mucosal mastocytosis (IL-9), eosinophilia (IL-5) (53), airway hyperreactivity and mucus hyperproduction (IL-13) (54), as well as isotype switching in IgG-producing B cells to allergen-specific IgE-producing cells (IL-4 and IL-13) (55). Moreover, the cytokine milieu stimulates eosinophils, mast cell maturation, and basophil recruitment (56).

Innate lymphoid cells type 2 (ILC2s) are also involved in the development of the pathology, as effector cells of airway inflammation in asthma (57). ILC2s express the GATA-3 transcription factor and produce IL-4, IL-5, IL-9, and IL-13 (58). The release of alarmins or lipid mediators such as PGD2 and cysteinyl leukotrienes (CysLTs), stimulates ILC2s to produce T_H_2 cytokines, such as IL-5 and IL-13, leading to increased recruitment of eosinophils in mucosal sites and exacerbating the inflammatory process (59, 60), thus contributing to the development of allergic asthma.

In the elicitation phase, re-exposure to the allergen will lead to its recognition and binding to mast cell-bound IgE. This interaction will induce the approximation of adjacent FcϵR1-IgE complexes, causing the activation of mast cells, leading to an early-phase reaction and causing an early-type bronchoconstrictor response (EAR) that lasts for 5–90 minutes (44). Upon activation, mast cells release a wide range of preformed inflammatory substances, such as histamine, neutral proteases, cytokines, and proteoglycans (61). Such substances will cause local or systemic symptoms, such as urticarial, flushing, vomiting, diarrhea, bronchospasm, rhinorrhea, and hypotension (62).

Hours after allergen-induced activation, mast cells *de novo* synthesize and release a wide range of proinflammatory and chemoattractant cytokines and inflammatory lipids, initiating the late-phase reaction (63). Cytokines, such as TNF-α, IL-5, and IL-10, can induce activation of DCs, T cells, and B cells (64). A consequence of this proinflammatory response, mainly mediated by TNF-α, is the margination and extravasation of T_H_2 cells, basophils, and eosinophils to the affected tissues (65). In addition, mast cells within the smooth muscle can be activated by allergen-IgE-FcϵR1 receptor interaction and release proinflammatory mediators that may facilitate AHR (66).

Eosinophils are recruited and activated by IL-3, IL-5, GM-CSF, and eotaxins (67). Activated eosinophils release several inflammatory mediators (human eosinophil major essential protein (MBP), eosinophil peroxidase, leukotrienes, IL-13, and TGF-β) causing airway constriction and AHR, goblet cell metaplasia, mucus overproduction, tissue damage, and airway remodeling (68–70). Lung eosinophilia is correlated with severe asthma, which has suggested that the number of eosinophils present in the airways could be a marker of the severity of the disease (71).

Over time, airway remodeling is a significant factor in the irreversible airflow obstruction and reduction of lung functionality observed in severe cases of asthma (72). Eosinophils and mast cells are responsible for these effects in the context of asthmatic disease, by infiltrating into tissue in response to cytokines produced by T_H_2 cells, as mentioned previously (73). Allergen-IgE complexes stimulate mast cells to produce a large variety of *de novo* synthesized and granularly stored mediators, including histamine, proteoglycans, proteases, prostaglandins and leukotrienes; cytokines such as IL-1β, IL-6, IL-13, and TNF-α; as well as chemokines and different growth factors (74). This also occurs in eosinophils, which release IL-3, IL-5, GM-CSF and eotaxins, among other proinflammatory mediators, inducing the thickening of airway walls, changes in the protein composition of the extracellular matrix, vascular leakage, goblet cells hyperplasia, mucus hypersecretion, and bronchial hyperresponsiveness (72). Therefore, these cells contribute significantly not only to immediate hypersensitivity and late-phase inflammation, but also to tissue remodeling in the airways.

If exposure to the allergen is repetitive over time, the proinflammatory milieu will persist and lead to a chronic allergic inflammation, characterized by a persistent type 2 immune response caused by the activation of T_H_2 lymphocytes, eosinophils, basophils, and macrophages (75). This chronic airway inflammation —a cardinal marker of asthma- results in parenchymal damage and a continuous process of repairing by generating connective tissue, aiming to preserve the optimal functionality of the airway, mainly through the reduction of airway hyperreactivity, given that stiffer airways may constrict less well in response to a stimulus than a thinner-walled airway (76, 77).

Despite the fact that most of the cases of allergic asthma present the aforementioned immunopathologic events, clinical immunologists have considered that such characterization is an oversimplification of the disease. This has led to a paradigm shift regarding the characteristics of the disease, on which allergic asthma is now classified into different endotypes, particularly type 2-high or ultra-high asthma, which is characterized by the increased number and function of pulmonary eosinophils, and type 2-low (also denominated non-type 2) asthma (59). Although this current review mainly focuses on type 2-high or eosinophilic allergic asthma and therapies for this particular endotype, it is worth to mention that type 2-low asthma is characterized by the absence of T_H_2 cytokines, being associated with later onset of the pathology, use of elevated dose of corticosteroids, and obesity (78). Furthermore, type 2-low asthma presents an elevated number of pulmonary neutrophils, increased levels of IL-6, and activation of the inflammasome pathway. More detailed information regarding the mechanism associated with non-type 2 asthma and treatment options has been reviewed on different articles (79–81).

## 3 NKT cells: a multiway bridge between innate and adaptive immunity

Since different subsets of immune cells are involved in the development of asthma, novel approaches that target other groups of cells, such as NKT cells, could improve current immunotherapies and lead to the development of novel therapeutic strategies with the potential to become the first curative approximation (82).

NKT cells are a highly conserved, non-conventional T cells subpopulation that participates in innate and adaptive immune responses by rapidly secreting various cytokines, which faculty these cells to exert immunomodulatory functions in different contexts, including tumor response, infectious diseases, allograft rejection, and autoimmune diseases (83, 84). As the name implies, these cells express cell-surface molecules from conventional T cells, such as T-cell receptors (TCR)-CD3 complex, and NK cells, such as CD161 (NK1.1 in mice), NKG2D, and proteins associated with the Ly49 receptor family (85, 86). The development of NKT cells begins in the thymus, where CD4^+^CD8^+^ double-positive (DP) thymocytes are selected based on whether the TCR recognizes self- or foreign lipids in the context of CD1d molecules (87). Then, selected cells further differentiate and finally migrate to peripheral locations, such as the liver, spleen, gut, and lungs (88).

Different types of NKT cells have been identified, and the current classification of NKT cell subsets is based on their phenotype (89). Type I NKT cells, also known as invariant NKT (iNKT) cells, are defined by an invariant TCRα chain expression (Vα14Jα18 in mice and Vα24Jα18 in humans) paired with a limited TCRβ chains repertoire (Vβ8, Vβ7, Vβ2 in mice and Vβ811 in humans) (90). Although more elusive, type II NKT cells, designated as diverse NKT (dNKT) cells, express a more diverse repertoire of TCRα and β chains, which enable the recognition of a wide range of self- and foreign lipid antigens also presented by CD1d (91). A distinctive property of dNKT cells is the null reactivity toward glycolipids recognized by iNKT cells, notwithstanding its capacity to become activated by compounds such as sulfatide, phosphatidylinositol, phosphatidylglycerol and β-GalCer (92). The complete identification and characterization of dNKT cells are still challenging due to technical limitations and the lack of specific markers (93). Because of this, most of the research has focused on studying the biology of iNKT cells, which will be the focus of this review.

In contrast to conventional T cells, iNKT cells become activated, mainly, by the recognition of glycolipid antigens bound by their tails to non-classical and non-polymorphic MHC class I-like CD1d glycoprotein (94), which is highly expressed on professional antigen-presenting cells (95, 96). Subsequent to its activation, NKT cells secrete copious amounts of various cytokines, including T_H_1-like (IFN-γ, TNF-α), T_H_2-like (IL-4, IL-6, IL-13), T_H_17-like (IL-17A, IL-22) and regulatory cytokines (IL-10) (97–100). This event determines the capacity of iNKT cells to stimulate and modulate the function of other immune cells, such as CD4^+^ and CD8^+^ T cells, B cells, DCs, and NK cells, via non-direct activation, also known as transactivation. Thus, iNKT cells are a functional bridge between innate and adaptive immunity, being capable of modifying the outcome of the immune response.

Interestingly, the type of cytokine secreted by iNKT cells depends on the expression of NK1.1 (101) and specific transcription factors associated with T cell differentiation, such as T-bet, GATA-3, RORγt, and PLZF (102). Thus, iNKT cells are classified according to the expression of these transcription factors: NKT1 (PLZF^lo^T-bet^+^), NKT2 (PLZF^hi^GATA-3^hi^), NKT10 (PLZF^lo^E4BP4^+^), and NKT17 (PLZF^int^RORγt^+^) (103, 104). Further analysis of these subsets allowed the establishment of the principal cytokines that these subsets secrete: IFN-γ (NKT1), IL-4 (NKT2), IL-10 (NKT10), and IL-17A (NKT17) (103).

A breakthrough in the study of iNKT cells was the discovery that virtually all of them react to α-galactosylceramide (αGalCer), a glycolipid present in extracts of the marine sponge *Agelas mauritianus* (105, 106). Further development of fluorescent-labeled αGalCer-CD1d tetramers allowed the detection and quantitation of iNKT cells by flow cytometry (107), and their purification using fluorescent- and magnetic-activated cell sorting (FACS and MACS, respectively) methods (108). Moreover, the activation of iNKT cells *in vivo* by the administration of αGalCer leads to a rapid secretion of T_H_1 (IFN-γ and TNF-α) and T_H_2 (IL-4, IL-5, and IL-13) cytokines within a few hours after injection (109), allowing the transactivation of innate and adaptive immune cells (110, 111).

### 3.1 Outlining the role of iNKT cells in asthmatic disease on animal models

It has been proposed that iNKT cells deploy a protective role in several pathologic conditions, such as tumors and some infectious and autoimmune diseases. Notwithstanding the previous role, these cells have been associated with pathogenic roles in other diseases, such as atherosclerosis, tissue transplant rejection, certain liver diseases, airway hyperresponsiveness, and asthma (112).

The first study addressing the possible pathogenic role of NKT cells in asthma revealed that the depletion of NK1.1^+^ cells, such as NK and NKT cells, before the immunization with ovalbumin (OVA) as a model of allergic asthma led to a reduction of airway eosinophilia and T cell infiltration in the lungs together with diminished levels of allergen-specific IgE (113). However, in CD1d1 mutant mice, characterized by reduced frequencies of NKT cells, the induction of OVA-specific allergic asthma resulted in pulmonary eosinophilic inflammation similar to that observed in wild-type mice, concluding that NKT cells were dispensable for allergen-induced asthma (113).

In contrast to earlier findings, Akbari et al. (2003) used *Cd1d^−/−^* and *Ja281* ^−/−^ mice (both strains lacking iNKT cells) to establish an allergic asthma model, observing a reduced airway eosinophilia and diminished OVA-specific IgE production, without development of AHR (114). These results implied that pulmonary NKT cells became activated soon after antigen encounters in the lungs and that these cells were required to induce AHR by secreting IL-4 and IL-13. Furthermore, using OVA-sensitized and challenged Jα18^−/−^ mice, Lisbonne et al. (2003) demonstrated that the absence of iNKT cells leads to a diminished AHR, reduced number of total cell number present in BAL fluid, and lower anti-OVA IgE titer (14). Later, it was demonstrated that activation of iNKT cells by intranasal administration of αGalCer in BALB/c mice was enough to induce asthma-related symptoms, including AHR and airway inflammation (115). Moreover, nasal administration of SP-30, a synthetic α-glucuronosylceramide derived from *Sphingomonas capsulata*, led to the induction of AHR, pulmonary eosinophilia, and increased serum IgE levels, being an independent response of other immune cells such as T cells, B cells, and eosinophils (115).

In 2011, Wingender et al. demonstrated that house dust extracts (HDE), considered as T_H_2-biasing mucosal adjuvants, could induce activation of DN3A4-1.2 cells, a well-characterized murine iNKT cell hybridoma, in a CD1d- and TCR-dependent manner, which suggests that HDE may contain antigens that are recognized by iNKT cells (116). HDE has also activated the human iNKT cell line, implying a similar response in both cell lines toward the same antigens. Furthermore, in order to evaluate the contribution of iNKT cells to the T_H_2-related activity of HDE, BALB/c wild-type and Jα18^−/−^ mice -which lack of iNKT cells- were immunized with OVA plus HDE, to be further airway challenged with OVA. Notably, immunization and airway challenge of wild-type mice led to the development of an eosinophilic airway inflammatory response, with elevated levels of T_H_2 cytokines and IgE responses, in opposition to the effects observed in the Jα18^−/−^ immunized mice, that although presented signs of allergen-induced airway inflammation -mainly due to the effects of HDE-, it was significantly lower in comparison to inflammation observed in wild-type mice. These results led to the proposition that iNKT cells were involved in the development of allergic asthma, most likely due to the secretion of cytokines derived from activated iNKT cells, given by the recognition of antigens present in HDE. Furthermore, Albaker et al. (2013) reported that the glycosphingolipid asperamide B, purified from *Aspergillus fumigatus* -a saprophytic fungus whose spores are highly recovered from soils and other environments- induced the activation of iNKT cells and caused AHR after treatment with a single intranasal dose (117). These results suggested that iNKT cell-induced asthma would depend on environmental exposure to air pollutants such as pollen particles, HDE, and fungal compounds. Other investigations have required the coadministration of αGalCer to induce allergic asthma, failing to induce the disease only by the administration of OVA or ragweed, suggesting that NKT cells, antigen-specific T_H_2 cells, and IL-4 were needed for the development of asthma, respectively (118, 119).

Notwithstanding the proposition that the secretion of specific cytokines derived from iNKT cells could promote the development of allergic asthma, it has also been proposed that iNKT cells could modify the immune landscape, particularly by counteracting the tolerogenic effects of T regulatory (T_reg_) cells, which are associated with the resolution of asthmatic inflammation and protection against experimental asthma. This was initially addressed by Thorburn and colleagues (120) on which immunoregulatory components derived from *Streptococcus pneumoniae*, namely, type-3-polysaccharide (T3P) and pneumolysoid (Ply), jointly reduce the number of eosinophils present in an OVA-induced allergic asthma murine model. Furthermore, the concomitant administration of T3P and Ply led to an increase in the number of pulmonary T_reg_ cells, which had the capacity to suppress the accumulation of NKT cells in the lungs and NKT cell-induced AHR, proposing that cell contact-mediated suppression was the main mechanism for this event. Later, Lu and collaborators (121) demonstrated that increased expression of Foxp3 on T_reg_ cells by the injection of lentiviral particles carrying the Foxp3 gene in an OVA-induced allergic asthma model caused the reduction of pulmonary NKT cells. Furthermore, intraperitoneal administration of α-GalCer in the same model led to increased percentage of pulmonary NKT while reducing the levels of T_reg_ present in lungs. Such results suggest a negative regulation between these cellular subsets, on which T_reg_ cells could have regulatory properties over NKT cells, blocking their activity with the concomitant reduction of allergic asthma symptoms. Furthermore, a recent study using a murine model for asthma demonstrated that enhancing the suppressive capabilities of T_reg_ cells through CD39 overexpression led to a lower number of pulmonary NKT cells, along with a reduced secretion of IL-4 and IFN-γ derived from these cells, causing a reduction in airway resistance, lower pulmonary eosinophilia, and reduced goblet cell hyperplasia (122). However, evidence presented by Chen et al. (123) has called into question the previously depicted counter-regulation between NKT and T_reg_ cells in the context of asthma, mainly given by the expansion of lung T_reg_ cells in *wild-type* mice but not in iNKT cell-knockout mice through the intraperitoneal administration of αGalCer. In particular, the αGalCer administration enhanced the secretion of IL-2 by iNKT cells, and the neutralization of this cytokine reduced the expansion of T_reg_ cells *in vivo* and *in vitro*. Thus, the authors suggested that the release of IL-2 by αGalCer-activated iNKT cells can induce the generation of lung T_reg_ cells in mice. Additionally, the same group demonstrated that intraperitoneal administration of α-GalCer previous to OVA sensitization caused a lower infiltration of inflammatory cells in the respiratory tract, reduced number of goblet cells in the airway epithelium, and lower number of eosinophils on bronchoalveolar-lavage fluid, as well as promoting the expansion and increased function of T_reg_ cells (124). Based on this evidence, the authors proposed that the production of IL-2 by αGalCer-activated iNKT cells was fundamental to promote the expansion of T_reg_ cells.

Given these facts, the exact mechanisms of the cross-regulation between NKT and T_reg_ cells are still debatable. It is important to note that further discussion about the relationship between iNKT cells and T_reg_ cells should consider the route of administration of glycolipids and the timing related to allergen sensitization, as well as evaluating other types of glycolipids that could be determinant in the interaction between NKT and T_reg_ cells and further depicting the molecular mechanisms by which such cross-regulations occurs.

The current classification of iNKT cells considers the existence of different subsets, which could explain their protective or pathological role in certain diseases (103). In the context of asthma, Kim et al. (2009), employing a T-bet^−/−^ murine model (125), suggested that even in reduced number, the remaining iNKT cells are sufficient for developing AHR, either induced by administration of αGalCer or OVA. A particular characteristic of this model is the significant reduction of IFN-γ with an increased IL-4 production, which could be associated with a predominance of iNKT2 cells, leading to an enhanced susceptibility to generate a T_H_2-biased response. Following this hypothesis, nasal administration of IL-25 in mice caused AHR and enhanced secretion of IL-4 and IL-13 due to higher expression of IL-17RB, a receptor of IL-25, associated with the phenotype of iNKT2 cells (126). In a recent report, Tumes et al. (2019) indicated that mice lacking the epigenetic regulatory enzyme enhancer of zest homolog 2 (Ezh2) presented an increased number of iNKT2 cells in association with higher levels of IgE, airway inflammation, and induced or spontaneous AHR, supporting the pathologic role of iNKT2 cells in the development of asthma (127). Considering these studies, iNKT cells could directly cause AHR and T_H_2 inflammation, outlining iNKT cells as a multifaceted subset in asthma by either fulfilling an adjuvant function or directly inducing AHR.

Humanized mouse models have been proposed to study the complexity of the immune response subsiding allergic diseases, avoiding the ethical constraints inherent to function studies on humans (128). In the context of the plausible pathogenic role of NKT cells in allergic asthma, Ose et al. (2021) evidenced that a challenge with either birch or grass pollen allergens on NSG mice that had received CD56-depleted PBMCs obtained from highly sensitized donors led to diminished production of allergen-specific IgE and reduced lung inflammation (129). Furthermore, pathologic airway resistance level and goblet cell hyperplasia were restored when NSG-SGM3 mice expressing human IL-3, GM-CSF, and stem cell factor received CD56-depleted PBMCs concomitantly with positively selected CD3^+^CD56^+^ iNKT cells.

### 3.2 Potential protective role of iNKT cells in allergic asthma models

Although all the aforementioned studies suggest that iNKT cells have a pathological role in the onset of asthma, other studies have reported that iNKT cells are unrelated to the development of allergic asthma. Matsuda et al. (2005) and Hachem et al. (2005) demonstrated that experimentally induced allergic asthma could be modulated by injection of αGalCer in OVA-sensitized mice previous a further OVA challenge, reducing the amount of pulmonary eosinophilic infiltration and AHR (130, 131). This suggests that αGalCer-induced secretion of IFN-γ could function protectively regarding the development of asthma by modulating the cytokine secretion from a T_H_2- towards a T_H_1-profile.

Koh et al. (2010) suggested that NKT cells are dispensable for developing chronic asthma (132). This research group also evaluated different allergic asthma hallmarks, such as airway remodeling characteristics, AHR, and eosinophilic airway inflammation. It was established that OVA-induced allergic asthma on BALB/c *Cd1d* ^−/−^ mice presented a significant increase in AHR, a higher number of total cells in BAL fluid, enhanced mucus metaplasia, subepithelial fibrosis, and smooth muscle hyperplasia with increased levels of IL-4 and IL-13 (132). These results could also imply that the pathogenesis of acute AHR –in which NKT cells have been reported to be fundamental in its onset- might differ from chronic AHR.

Applying a protocol for allergic asthma induction in response to OVA on a triple-knockout murine model that presented only NKT cells and activated CD8^+^ T cells, Das et al. (2006) reported that while wild-type mice developed eosinophilic airway inflammation in conjunction with increased levels of IL-4 and IL-5 in BAL, *H2-K^b−/−^H2-D^b−/−^C2ta^−/−^* triple-knockout mice did not develop airway allergic inflammation (15). This evidence allowed them to suggest that NKT cells and activated CD8^+^ T cells were not sufficient to induce symptoms associated with the onset of asthma. Furthermore, depleting NKT cells from wild-type BALB/c and C57BL/6 mice did not avoid the induction of allergic asthma, suggesting again that NKT cells are dispensable for establishing this disease.

McKnight et al. (2017) found no significant difference among different models of induced airway disease when comparing wild-type and iNKT cell-deficient mice concerning the number of lung iNKT cells present after challenge, the onset of AHR, and severity after administration of anti-CD1d monoclonal antibody (133). Based on these results, the authors proposed that the difference between the murine models employed to study the role of iNKT cells in asthma could be influenced by the microbiota the animals are exposed to.

Chang et al. (2011) showed that influenza virus A (H3N1) infection in suckling but not in adult mice could induce a protective effect by CD4^-^CD8^-^ (DN) NKT cells in a T-bet TLR7-dependent manner (134). The authors associated the protective effect with the maturation and expansion of DN NKT cells, which might have led to the expansion of T_reg_ cells. Furthermore, administration of iNKT cells ligands such as αGalCer or antigens derived from *Helicobacter pylori* replicated the protective effect of NKT cells, as seen in influenza virus infection. This was the first study to propose a subset of NKT cells that could suppress AHR in conjunction to provide a mechanism for the hygiene hypothesis, therefore proposing a possible therapeutic approximation by using compounds derived from microorganisms in an NKT cell-based strategy. Further studies of the DN NKT cell population revealed a high expression of CD38, which could suppress CD4^+^ T cells through cytotoxic activity and prevent the development of AHR (135).

Further investigations have developed multiple αGalCer-derived glycolipid analogs that can induce a biased T_H_1/T_H_2 cytokine response (136–138). It has been reported that the co-administration of αGalCer-modified analog α-lactosylceramide (αLacCer) -a weak activator of murine and human iNKT cells- and αGalCer led to a reduced airway hyperreactivity and neutrophil infiltration, accompanied by lower production of IL-4 and IL-13, in comparison to the basal levels induced by the administration of αGalCer alone (139).

Recent evidence has called into question the previously depicted counter-regulation between NKT and Treg cells in asthma. In 2019, Chen et al. reported that intraperitoneal administration of αGalCer promotes the expansion of lung T_reg_ cells in WT mice but not in iNKT cell-knockout mice. In particular, the αGalCer administration enhanced the secretion of IL-2 by iNKT cells, and the neutralization of this cytokine reduced the expansion of T_reg_ cells *in vivo* and *in vitro*. Thus, the authors suggested that the release of IL-2 by αGalCer-activated iNKT cells can induce the generation of lung T_reg_ cells in mice (123). Later that year, the same research group reported the expansion and increased suppressive activity of T_reg_ cells within pulmonary tissue on wild-type BALB/c mice that received a single dose of αGalCer previous to allergen sensitization, leading to decreased T_H_2 immune response (124). Given these facts, the exact mechanisms of the cross-regulation between NKT and T_reg_ cells are still debatable. It is important to note that further discussion about the relationship between iNKT cells and T_reg_ cells should consider the route of administration of glycolipids and the timing related to allergen sensitization.

### 3.3 Conflicting evidence regarding the presence and activity of NKT cells in asthmatic patients

Murine models of asthma have been of significant importance for studying the possible role of NKT cells in this disease; however, mice do not reproduce exactly the pathological state evidenced in humans with asthma, including differences in the degree of symptoms and events associated with the chronicity of the disease, such as airway remodeling, and the use of compounds employed to induce experimental allergic asthma, which are generally innocuous to humans, particularly regarding the extended use of OVA as a model allergen in murine models.

The first approximation to determine the possible role of NKT cells in asthmatic patients evaluated the frequency of these cells in peripheral blood. Interestingly, asthmatic patients showed a lower NKT cell count than healthy controls. Besides, there was no correlation between the number of NKT cells and clinical variables, such as eosinophil count and serum IgE level, among others (140).

Conversely, other studies have reported elevated frequency of iNKT cells in BAL fluid from asthmatic patients compared to healthy controls, in addition to a reduced number of iNKT cells in peripheral blood, which might suggest a process of migration of these cells from the periphery to the airways (141–143).

Moreover, Agea et al. (2005) reported that human T cells, including iNKT cells, may recognize lipids from pollens –particularly phosphatidylcholine and phosphatidylethanolamines- through a CD1-dependent pathway, requiring CD1a^+^ and CD1d^+^ antigen presenting cell (144).

An initial report by Akbari et al. (2006) showed that more than 60% of pulmonary CD4^+^CD3^+^ T cells present in BAL fluid from patients with moderate or severe asthma were iNKT cells (145). However, other groups have failed to replicate the same results, evidencing that the presence of iNKT cells in BAL fluid, induced sputum, and bronchial-biopsy specimens ranges from 0.07% to 3% (141, 142, 146). Furthermore, the study conducted by Vijayanand and collaborators employing different lung-derived samples was unable to observe the results reported by Akbari et al., on which less than 2% of NKT cells were detected on pulmonary samples and were unable to detect the NKT T-cell receptor genes Vα24 and Vβ11 on bronchoalveolar-lavage fluid and sputum of asthmatic subjects (147). In this sense, it has been suggested that the results obtained by Akbari et al. (2006) may be biased due to improper gating strategy and lack of blocking Fc receptors that could have led to nonspecifically binding of antibodies (143).

Although limited by the small number of patients, the results presented by Reynolds et al. (2009) evidence an increased presence of iNKT cells in lung biopsies of patients with mild-to-moderate asthma taken at baseline, 24 hours and seven days after allergen challenge, similar context to mouse models previously used (148). By using αGalCer-loaded CD1d tetramers, the group reported that 9.8% of CD3^+^ T cells were iNKT cells at baseline, increasing 24 hours after the allergen challenge to 15%, returning to baseline levels after seven days. By correlating the results with the measurement of AHR by spirometry, the group proposed that iNKT cells would have a crucial role in allergic asthma by increasing AHR, being the first study to recreate similar conditions as those studies employing murine models.

Furthermore, the presence of iNKT cells in other types of samples, such as induced sputum, has been determined to be increased in patients with asthma and eosinophilic bronchitis (149). The same study also found an inverse correlation between the number of iNKT cells in sputum and the degree of AHR, proposing that specific cytokines produced by iNKT cells, such as IFN-γ, could inhibit AHR. In a further study, the same research group assessed the cytokine produced by iNKT cells present in the blood of asthmatic patients, evidencing an enhanced production of IL-4, which may contribute to the inflammatory process in the airways and the severity of the diseases (150).

Carpio-Pedroza et al. (2013) found an increased frequency of iNKT cells in peripheral blood during asthma exacerbation attacks in children (151). Even more, this study showed that iNKT cells could be influencing asthmatic exacerbations due to increased production of IL-4 and decreased levels of IFN-γ, proposing that iNKT cells could modulate these episodes by the polarization of T cells and recruitment of pro-inflammatory cells. These results align with the previous work by Yan-ming et al. (2012), where they evidence an increased production of IL-4 by iNKT cells (152). This study also demonstrated a reduction of IL-4 in sublingual immunotherapy treatment for house dust mite allergy with no further increase in IFN-γ levels. However, it enhanced the production of IL-10, suggesting a possible mechanism of immunotherapy through immune tolerance induction.

Adding to the controversy regarding the role of iNKT cells in asthmatic patients, it has been depicted that the aforementioned cells could interact either synergistically or antagonistically with T_reg_ cells, mainly through the secretion of IL-2 from iNKT cells, causing an increased proliferation of T_reg_ cells (153), or the suppression of the proliferation and cytokine secretion of NKT cells by T_reg_ cells (154), respectively. Nguyen et al. (155) explored such interaction on samples derived from allergic asthma patients, evidencing an increased expression of natural cytotoxic receptors NKp30 and NKp46 on iNKT cells from patients with allergic asthma, as well as an elevated secretion of granzyme B and perforin by these cells, which led to an increased cytotoxicity of iNKT cells against autologous Treg cells, suggesting that the reduction of T_reg_ cells caused by iNKT cells could be mediated either by the direct interaction or through the secretion of the aforementioned cytotoxic enzymes. Such results suggest that iNKT cells may also contribute to the pathogenesis of AHR by acting as counter-regulators of T_reg_ cells.

In the context of the usage of bacterial lysate, such as OM-85 Broncho-Vaxom (OM-85 BV), as a clinical immunomodulatory therapy, Lu et al. (2015) demonstrated a significant increase in the number of peripheral blood iNKT cells in asthmatic children treated with OM-85 BV, further evidencing a decreased production of IL-4 and enhanced secretion of IL-10 from these cells. This evidence suggests that therapeutic strategies-based modulation of the immune response by iNKT cells could induce allergen-specific tolerance and a possible curative therapy in the context of asthmatic disease.

### 3.4 Methodological differences regarding studies of iNKT cells involvement in asthma

Given the conflicting results observed in both murine model and samples derived from asthmatic patients, measures should be taken to properly evaluate the contribution of NKT cells in the onset or severity of allergic asthma, among which should consider the following aspects:

#### 3.4.1 Mice strain background

It is a well-established fact that the differences in genetic background of mice determine inflammatory characteristics, being critical for the development of relevant murine models of allergic asthma. It has been reported that A/J and AKR/J mice present higher levels of AHR after allergen sensitization and challenge (156). However, the mouse strain most frequently employed for allergic asthma studies are C57BL/6 and BALB/c. Comparative studies have indicated that BALB/c mice are prone to developing a T_H_2 response and developing AHR, while C57BL/6 mice are hyporesponsive to methacholine challenges although displaying a considerable allergen-induced eosinophilic inflammation (157).

#### 3.4.2 Animal models to determine the significance of NKT cells in asthma

Difference between models that consider either the deletion of critical genes for developing NKT cells, deletion of CD1d genes, or administration of blocking antibodies should weigh whether such approaches effectively allow evaluating the participation of NKT cells on allergic asthma, taking into consideration the different subtypes of NKT cells that exist. Some models, such as Jα18^−/−^, still endows the development of dNKT cells, which could be further stimulated by unidentified antigens and/or mechanisms that could modulate the onset of asthma (158).

#### 3.4.3 Microbe exposure

The current microbiota present in the subject could be conditioning the immune response in the development of asthmatic diseases. The presence or absence of different bacteria would affect the immune response and inflammation (159). Furthermore, intestinal and mucosal microbiota could affect murine models employed to evaluate the participation of NKT cells in asthma (160, 161).

#### 3.4.4 Allergens

Particularly in protein-induced allergic asthma, endotoxin content in immunizing content could influence the development of asthma and the type of allergic response associated with the diseases (162, 163). Additionally, even though the use of OVA as a model allergen presents benefits, such as high accessibility, increased purity of the compound and the characterization of the epitopes against which immune responses are mounted, its use has been called into question, mainly due to the fact that inhalation of pure OVA induces a tolerogenic response (164), it is not an environmental allergen and does not cause airway inflammation in humans. Thus, the use of other allergens, such as ragweed, house dust mite (HDM) extracts and *Aspergillus fumigatus* (165–167) has been proposed in the development of model that resemble more closely to the allergic asthma observed in humans.

#### 3.4.5 Routes of sensitization and challenge

Different routes are employed depending on whether the objective is to induce acute or chronic asthma. One publication reported that subcutaneous sensitization was superior to intraperitoneal administration; however, is still pending to confirm this evidence (168). Furthermore, allergic responses in the murine asthma model do not resemble the natural induction of allergic disease, mainly because allergen exposure is a continuous event through time. In addition, current protocols have suggested chronic exposure to aeroallergens as a “physiological” approximation to the induction of allergic asthma (166, 169). In line with that knowledge, it has been recommended allergen exposure should consider inhalation by nebulization, intratracheal or intranasal administration (170–172).

#### 3.4.6 Proper identification of iNKT cells

Concise detection of iNKT cells should consider staining with fluorescent-labeled αGalCer-CD1d tetramer (173), as well as employing proper reagents to block unspecific interactions with other cells. Furthermore, iNKT cells could be activated by recognizing glycolipid content in the tetramer. This should be considered in adoptive transfer experiments, where iNKT cells could become transitorily anergic due to undesired activation (174), leading to a downregulation of the expression of its TCR. On the other hand, for human samples, as well as including tetramer staining, identification of NKT cells should also be complemented with the use of Vβ11, Vα24 and Vα24-Jα18-specific antibodies (147).

### 3.5 Reconsidering current treatment of asthma

Asthma is considered a heterogeneous disease on which different ‘asthma phenotypes’ have been recognized (175). According to the demographic, clinical and/or pathophysiological characteristics, the most common phenotypes of asthma include allergic asthma, non-allergic asthma, adult-onset asthma, asthma with persistent airflow limitation and asthma with obesity (175–177).

The current pharmacological therapy for asthmatic patients includes inhaled short-acting β-agonists (178), long-acting β-agonist (179), inhaled corticosteroids (180), systemic corticosteroids (181), leukotriene receptor antagonist (182), and biological agents, mainly monoclonal antibodies, directed against different immunological targets involved in the occurrence and severity of allergic asthma symptoms (183–186). Among the biological agents currently available for asthma treatment, some of the most used are mepolizumab, reslizumab, and benralizumab, which interfere with the functions of IL-5 and, in the case of the latter, has cytotoxic activity against cells that express the IL-5 receptor; dupilumab, that blocks IL-4 receptor α (187–189); omalizumab, that leads to a reduce binding of IgE to its receptor as well as downregulates the FcϵRI expression (190); and tezepelumab, that blocks TSLP and probed useful in cases of type-2 low asthma (191).

According to the severity of the disease, different therapeutic strategies can be considered. In the case of mild asthma, defined as well controlled asthma, treatment considers low-dose of inhaled corticosteroids and, when needed, short-acting β-agonists. On the other hand, treatment for moderate asthma, also considered as a well-controlled asthma, contemplates the use of medium-dose inhaled corticosteroids and long-acting β-agonist. Finally, severe asthma, defined as asthma that remains uncontrolled despite optimized treatment, considers the use of long-acting muscarinic antagonists, leukotriene receptor antagonist therapy and the use of biological agents, such as the aforementioned monoclonal antibodies (192).

However, high failure rates (30% to 70%) to adhere to the treatment regimen in asthma patients, as well as the high cost of treatment (193) and the heterogeneity in the immunopathology of the disease, impose significant limitations that can impair the effectiveness of the treatment, leading to the disappearance of therapeutic effects (10–12).

As current management of asthma considers only the regulation of asthmatic symptomatology, allergen desensitization immunotherapy arises as the unique treatment that can revert allergic diseases since it can suppress the proinflammatory state and promote the development of allergen tolerance (194).

Allergen immunotherapy leads to the generation of regulatory cells, such as regulatory T (Treg) cells and regulatory B (Breg) cells (195, 196), which produce inhibitory cytokines, such as IL-10 and TGF-β, as well as possessing specific molecules, such as granzyme B, CD39, CD73 and CTLA-4, that promote an immunosuppressive environment in the context of allergic inflammation (197, 198). Through these mechanisms, both Treg and Breg cells suppress allergic T_H_2 immune responses, as well as suppressing the production of allergen-specific IgE and inducing the secretion of IgG4 and IgA antibodies on B cells; abolish the homing of T_H_2 cells on inflamed tissues; suppress the activation of epithelial cells and mucus production; reduce the activation threshold of innate immune cells, such as mast cells, basophils and eosinophils; and interfere in the differentiation of naïve CD4^+^ T cells to T_H_2 cells (197, 199). Thus, the overall result of allergen immunotherapy leads to the generation of regulatory cells that suppress both T_H_1 and T_H_2 responses, to later promote a pronounced T_H_1 response to the administered allergen (200).

However, allergen immunotherapy has certain drawbacks that limit its use. Initially, candidates for allergen immunotherapy should present concise result of allergy testing, such as immediate hypersensitivity skin test or presence of serum specific IgE, while patients with positive test for specific IgE antibodies that do not correlate with clinical symptoms are not considered for the treatment (200). Secondly, previous to initiate allergen immunotherapy, patients should have a controlled asthma through the use of pharmacotherapy (200). Thirdly, allergen immunotherapy could induce adverse reactions, such as local allergic reactions, anaphylaxis, or near-death reactions (201). Fourthly, effectiveness of allergen immunotherapy mostly relies on the subjective assessment of the patient’s report of feeling better during a season that previously caused asthmatic symptoms (200). Furthermore, the immunotherapy build-up regime could last between 3 and 6 months, while the maintenance regimen could extend from 3 to 5 years (202). Additionally, discontinuation of allergen immunotherapy could lead to a relapse of the asthmatic symptomatology, reducing its effectiveness over extended periods (200).

Given these facts, improvements on allergen immunotherapy are required, mainly to induce an early tolerogenic response and prolonged effectiveness through time. In this way, allergen immunotherapy could benefit from multiple αGalCer-derived analogs capable of activating iNKT cells that have recently been developed in order to generate a biased T_H_1/T_H_2 cytokine response (203–205), and combined with different strategies designed to deliver the glycolipid analog *cargo*, such as the use of nanoparticles designed to induce the activation of iNKT cells (206), would allow joint delivery of both iNKT cells modulating glycolipids and allergens, allowing a possible restoring of the imbalanced cytokine production present in asthma by further induction of a tolerogenic immune response toward a specific allergen. Suzuki et al. (2019) led one distinguishable investigation that used αGalCer-loaded liposomes jointly delivered with OVA as a therapeutic strategy in a murine model of allergen-induced asthmatic disease, demonstrating a switch of immunoglobulins generated upregulation of T_H_1-type cytokine secretion and reversion on nasal symptoms (207).

On the other hand, an earlier immunosuppressive milieu could be established through the induction of NKT10 cells (100), which produce and secrete IL-10, which might lead to the generation of other cellular subsets producing such immunosuppressive cytokines, and could significantly impact the immune response in the context of allergic inflammation.

Finally, it has been demonstrated that activation of NKT cells can lead to the generation of Breg cells, as evidenced by Zeng (208) and Vomhof-DeKrey (209), which could enhance the generation of such cells in the context of allergen immunotherapy and promote a stronger anti-inflammatory response.

## 4 Concluding remarks

**Figure 4:**
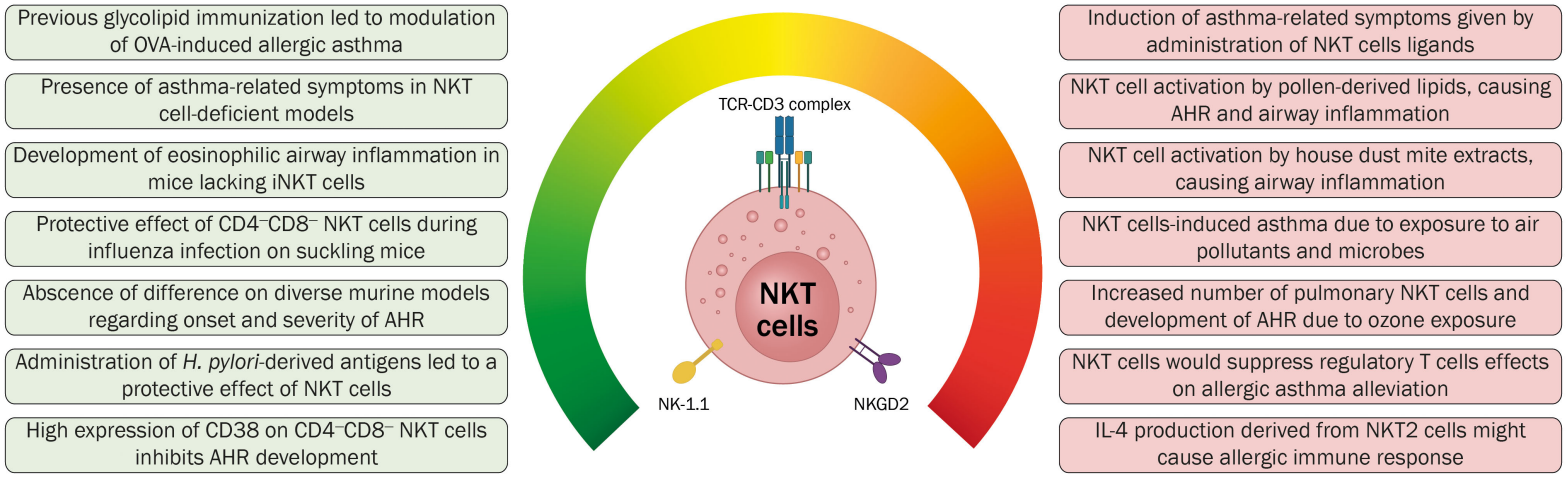
Contrasting evidence regarding the role of iNKT cells in the onset and development of allergic asthma. The results of different studies aiming to elucidate the function of this cellular immune subset have been inconclusive and contradictory. Further investigation regarding the role of iNKT cells in allergic asthma and the interaction of this cellular subset is required to generate novel immunotherapeutic strategies.

The information provided supports the fact that, in the context of allergic asthma, iNKT cells are present; however, their precise pathological or protective functions on this pathology remains unclear (**Figure 4**). Differences in the reported biological role of iNKT cells may also be due to biases as a result of methodological variations. Further studies employing murine models should take into account the proper genetic background of mice, the presence of iNKT cells in different types of tissues and samples retrieved from the animals, as well as the proper identification of the different subtypes of iNKT cells and the cytokine profile secreted by these cells. Regarding the participation of iNKT cells in human subjects, studies should consider the accurate identification of these cells employing either specific antibodies or CD1d tetramers, as well as proper staining protocols. Even more, it is pending evaluation if iNKT cells could participate in asthmatic disease caused by other etiologies, such as chronic, aspirin-induced, occupational, and steroid-resistant asthma.

Considering the capacity of iNKT cells to modulate different immune cell subsets, further investigations should focus on inducing the activation of these cells on asthmatic pulmonary tissue, promoting an anti-inflammatory cytokine milieu that would lead to the reduction of the symptomatology and, ultimately, to the reversion of the pathology. Future studies need to take into consideration the possible role that iNKT cells could be playing in the context of asthma to develop efficient immunotherapies that not only lead to the reversion of T_H_2-type cytokine overproduction but also generate a strategy that could be fully accomplished by the patient in a reduced regimen and leading to a lengthy tolerogenic response, arising as a time- and cost-effective therapy.

## Author contributions

CG-V: Conceptualization, Investigation, Writing – original draft, Writing – review & editing. RG: Writing – original draft, Writing – review & editing. PP: Writing – original draft, Writing – review & editing. MM: Writing – review & editing. DM: Writing – review & editing. CV: Writing – review & editing. FO: Writing – review & editing. FS: Funding acquisition, Writing – review & editing. AK: Funding acquisition, Supervision, Writing – review & editing. PG: Funding acquisition, Supervision, Writing – review & editing. NS-A: Funding acquisition, Supervision, Writing – review & editing. SP: Funding acquisition, Supervision, Validation, Writing – review & editing. LC: Funding acquisition, Supervision, Validation, Writing – original draft, Writing – review & editing.
